# The Multiple Etiologies of Abdominal Pain Post Roux-en-Y Gastric Bypass: A Case Series and Review of Management Strategies

**DOI:** 10.7759/cureus.34271

**Published:** 2023-01-27

**Authors:** Shaniah S Holder, Rosheyla Saint-Hilaire, Christopher Meusburger, Douvae Miller, Frederick Tiesenga

**Affiliations:** 1 Medicine, American University of Barbados School of Medicine, Bridgetown, BRB; 2 Medicine, St. George's University School of Medicine, True Blue, GRD; 3 Medicine, Saint James School of Medicine, Chicago, USA; 4 General Surgery, West Suburban Medical Center, Chicago, USA

**Keywords:** marginal ulcers, small bowel obstruction, intussusception, abdominal pain, gastric bypass, bariatric surgery

## Abstract

Roux-en-Y gastric bypass (RYGB) is one of the most common bariatric surgeries performed and has aided many people with their weight loss efforts. However, manipulating the gastrointestinal anatomy may have numerous consequences. Various complications of RYGB can occur during the early to late post-op periods, and abdominal pain is the most common symptom reported. The etiologies that present as abdominal pain are heterogeneous in anatomical origin, onset, severity, and management; therefore, differentiating diagnoses is crucial. The physical exam, inciting triggers, and alleviating factors can direct diagnostic measures accordingly. Prompt recognition and identification of these patients' underlying causes of abdominal pain are vital for accurate diagnosis and treatment. Methods can range from conservative management to surgical intervention, depending on the severity of the complication. In this report, we recount the cases of two patients who underwent elective RYGB and presented to the emergency department (ED) months later with abdominal pain. After the labs were taken and diagnostic tests were conducted, it was discovered that both patients had multiple underlying factors that could have contributed to their pain. This study aims to describe the diverse etiologies of abdominal pain encountered in these bariatric patients and explore the appropriate management strategies utilized for each case.

## Introduction

Obesity is a global epidemic with a 14% prevalence that is expected to rise to 20% by 2025 [1]. Obesity is accompanied by many comorbidities, such as cardiovascular disease and type 2 diabetes (DM-2), which can decrease a person's quality of life. Bariatric surgery is effective in the long-term management of obesity and its associated comorbidities. To this day, the most popular procedures performed include sleeve gastrectomy, Roux-en-Y gastric bypass (RYGB), and laparoscopic band placement [2]. Laparoscopic sleeve gastrectomy is the most common procedure done since it is less technically challenging than laparoscopic Roux-en-Y gastric bypass (RYGB) and does not require anastomosis creation or intestinal bypass [2]. RYGB is the second most commonly performed bariatric procedure in the United States after sleeve gastrectomy and is considered a safe procedure with a low mortality rate of 0.09 percent [3]. 

Despite its safety, RYGB is associated with many postoperative complications; therefore, it is essential to exhibit high clinical suspicion for swift diagnosis and effective management [3]. Approximately 15%-30% of patients have post-RYGB complications within the first three years, and due to the involvement of the gastrointestinal system, the most common symptom is abdominal pain [4,5]. There are many underlying etiologies of abdominal pain post-RYGB, ranging from overeating and acquired food intolerance to more complicated causes such as intussusception, ulcers, and obstruction [5]. Knowing the most common to least common complications associated with abdominal pain can assist doctors in determining the steps in management required to provide adequate care. Marginal ulcers are one of the most common complications and occur in 2%-15% of patients [5]. Small bowel obstruction is on the rare side, with an incidence of 0.2% to 1% [5]. Intussusception is the rarest, with an incidence of approximately 0.1% [5]. These complications are associated with abdominal pain in addition to fever, nausea, vomiting, diarrhea, constipation, and dysphagia [5].

In this report, we describe the cases of two patients who presented with abdominal pain months after elective RYGB surgery. After further testing with labs and diagnostic imaging, both cases were found to have multiple post-gastric bypass complications, with each one being a possible underlying etiology of their abdominal pain.

## Case presentation

Case one

**Figure 1:**
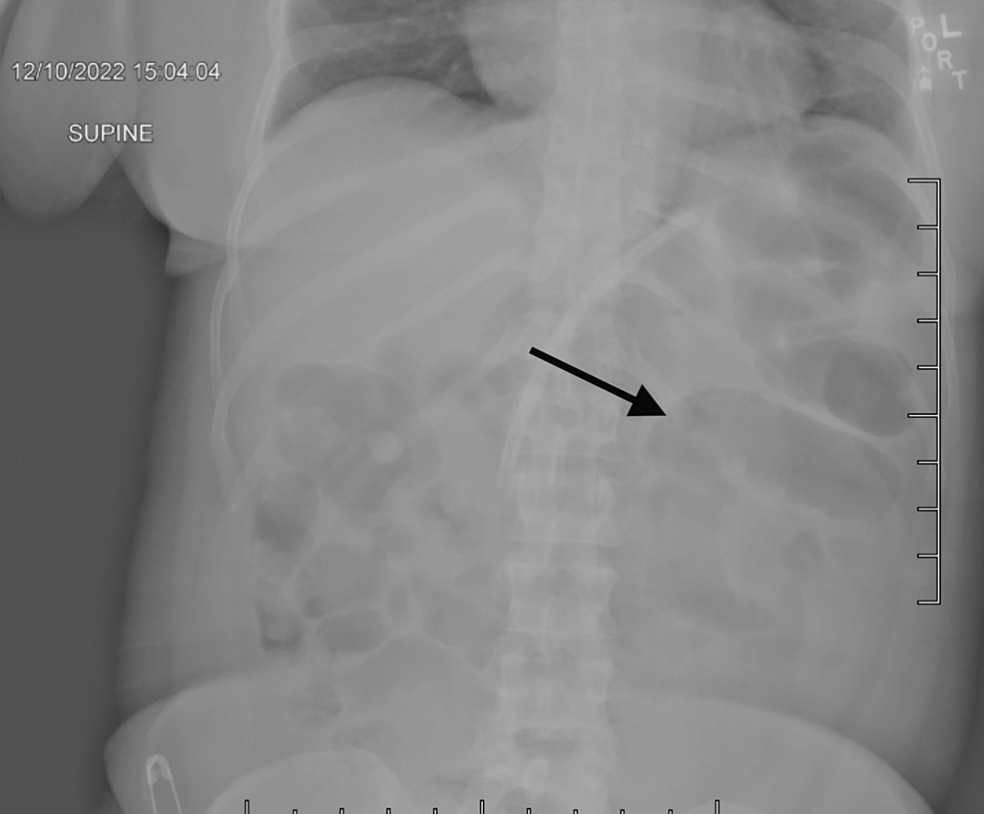
Abdominal X-ray with an arrow pointing to the dilated bowel loops

We present the case of a 40-year-old female patient with a body mass index (BMI) above 40 kg/m2. She underwent an elective laparoscopic Roux-en-Y gastric bypass, and following a normal postoperative period, she was subsequently discharged. Six weeks later, the patient presented to the emergency department (ED) with abdominal pain, obstipation, nausea, and vomiting. On physical exam, the abdomen was mildly distended and tender to palpation in the upper quadrants. An abdominal X-ray was conducted, and the results showed dilated small and large bowel loops with mild fecal burden, indicating early bowel obstruction. Figure 1 shows the dilated bowel loops.

The patient underwent an emergent diagnostic laparoscopy with adhesiolysis and small bowel release. During the surgery, multiple small bowel loops were dilated with a single adhesion visualized tethered to the gastrojejunostomy (GJ). The adhesion was lysed, and the obstruction appeared to be completely resolved. The Roux limb was followed from the stomach to the jejunojejunal (JJ) anastomosis and up to the Treitz ligament, then down to the ileocecal valve, to ensure no other adhesions or obstructions were present. The surgery was successful; after two days postoperatively, the patient had a bowel movement.

**Figure 2:**
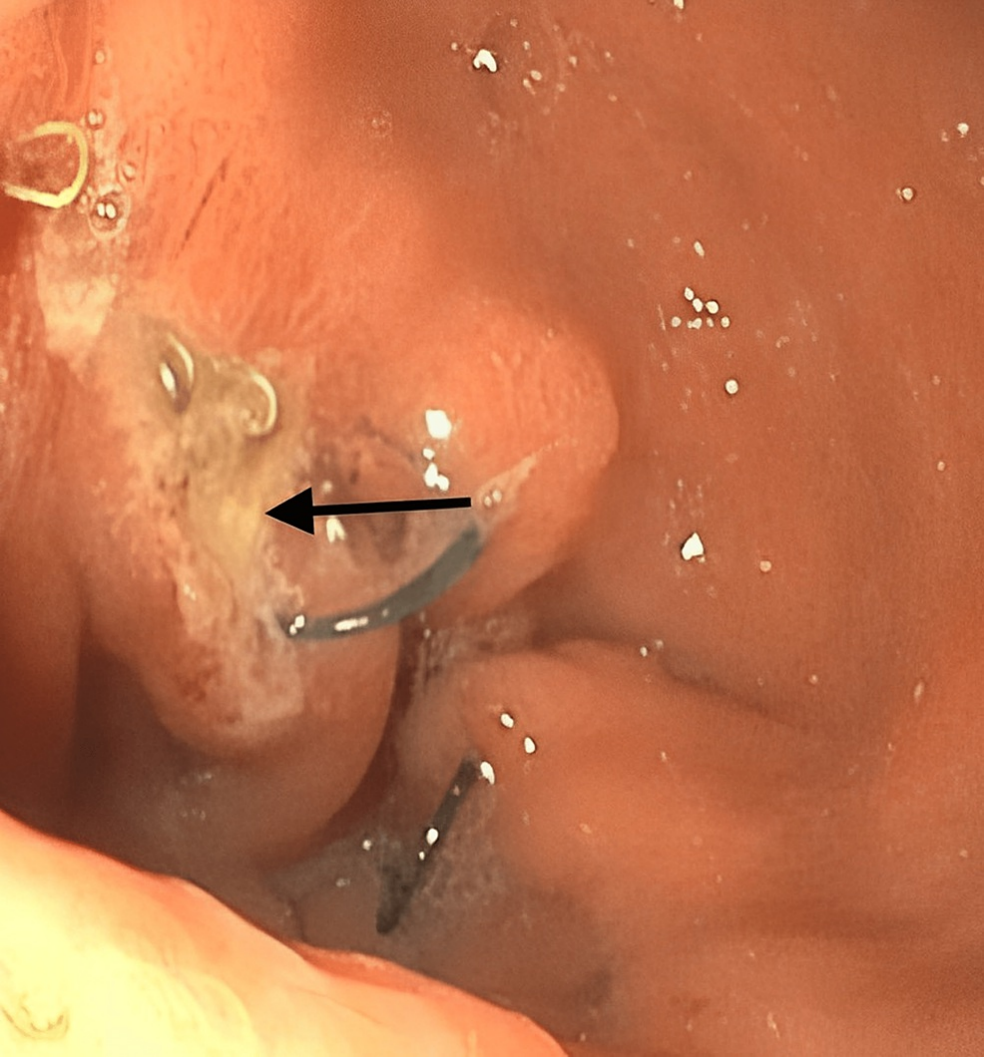
Endoscopic visualization of a marginal ulcer by the gastrojejunal anastomosis

Although her bowel function resumed, the patient still complained of continuous abdominal pain that was worse in the left upper quadrant (LUQ), along with persistent nausea and vomiting. Upon further questioning, it was found that the pain worsened after liquid intake; this was an indication for an esophagogastroduodenoscopy (EGD) for a suspected marginal ulcer. Located by the gastrojejunal anastomosis was a marginal ulcer, confirming the diagnosis. Figure 2 shows the positive EGD findings.

There was no perforation of the ulcer, so the patient began conservative management with a regimen of proton pump inhibitors (PPI) and acetaminophen for analgesia. Two days later, the patient endorsed complete relief of her symptoms and was instructed to follow up as needed.

Case two

**Figure 3:**
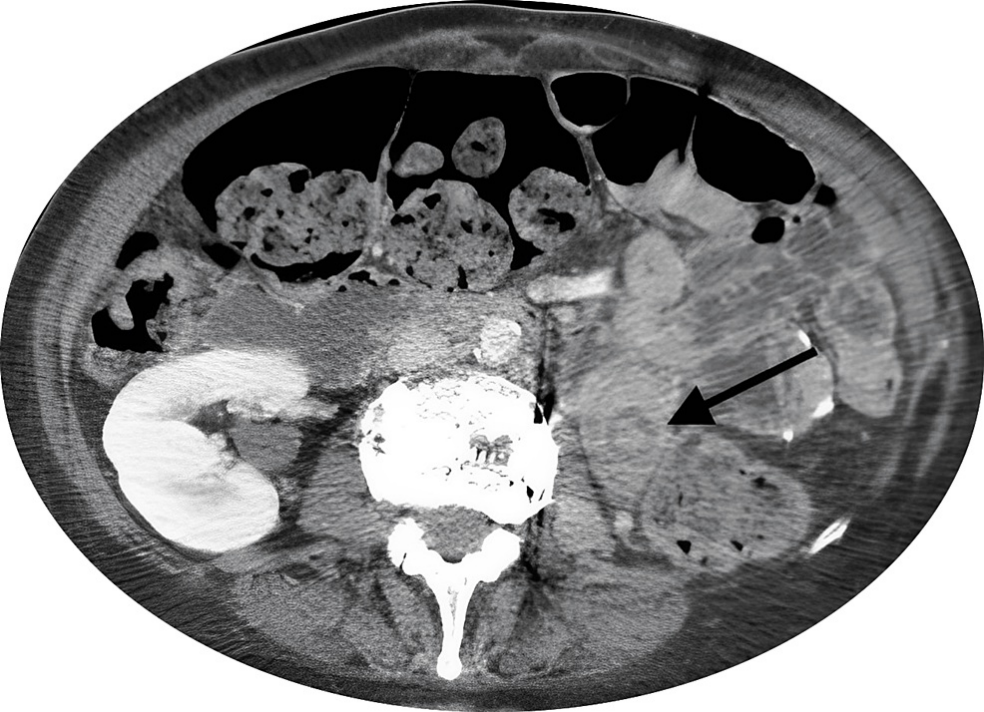
Arrow pointing to a target-like mass of bowel indicating intussusception

In this case, a 63-year-old female patient with a BMI of 47 kg/m2 underwent a Roux-en-Y gastric bypass. After an uncomplicated procedure with a normal postoperative period, the patient was discharged. Nine months later, after losing 36 kg, she presented to the ED with complaints of worsening upper abdominal pain and constipation that was refractory to medication and laxative use. She stated that her pain was 10/10, constant, and more severe with movement, and she denied vomiting or having bloody stools. On physical examination, there was diffuse tenderness that worsened on palpation. A computed tomography (CT) of the abdomen and pelvis with intravenous contrast was conducted and showed an eccentric area of hypoattenuation within the small bowel, representing a segment of intussusception that extended approximately 6 cm. Figure 3 highlights the intussusception found on CT with an arrow pointing to the mass.

**Figure 4:**
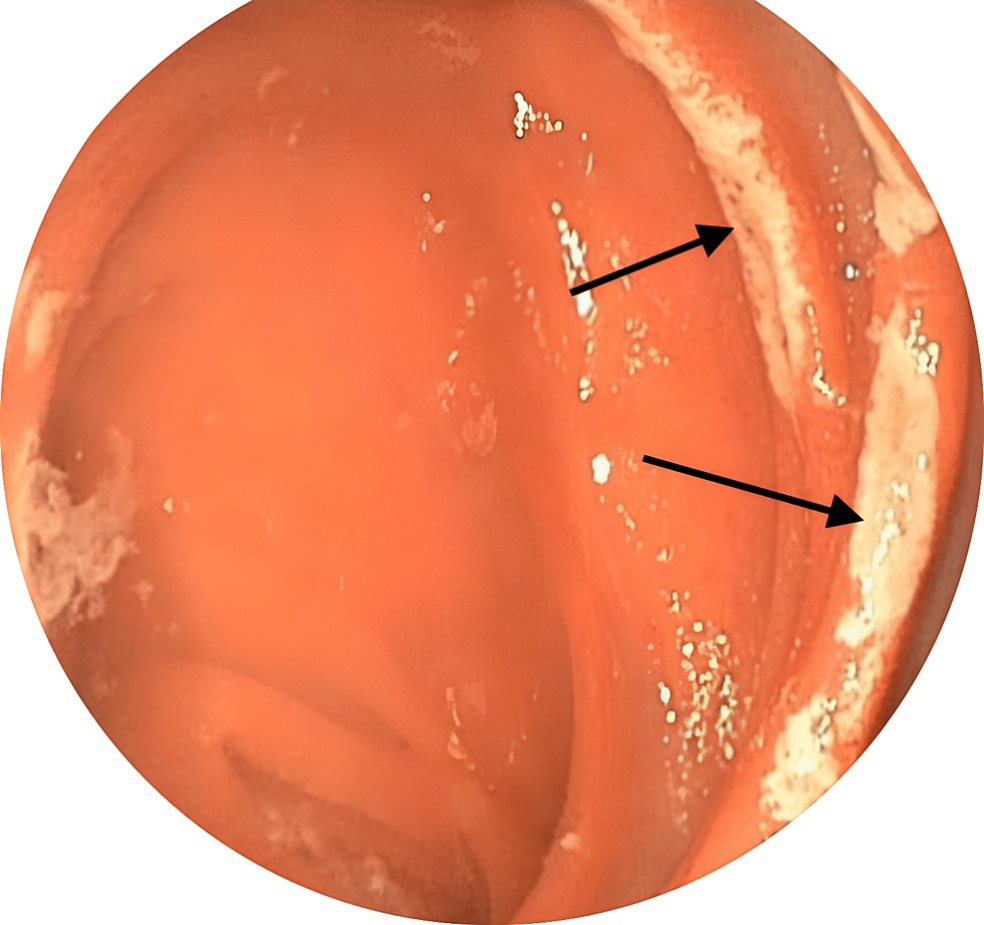
An endoscopic view of multiple marginal ulcers located in the jejunal mucosa distal to the gastrojejunal anastomosis

The patient underwent an emergent diagnostic laparoscopy, and the intussusception was found just beyond the JJ anastomosis. It was easily reducible without an event and appeared to be intermittently telescoping into itself, as there was no lead point found. After the surgery, the patient had a bowel movement but still complained of pain with focal tenderness to palpation in the epigastric region. An EGD was conducted due to the suspicion of marginal ulcer formation and a significant finding of multiple ulcers in the jejunal mucosa distal to the GJ anastomosis. Figure 4 shows the marginal ulcers found on EGD.

A regimen of PPI, antiemetics, and analgesics was initiated following the discovery. The patient endorsed the relief of her symptoms and was subsequently discharged with a plan to follow up.

## Discussion

Roux-En-Y Gastric Bypass is characterized by the creation of a gastric pouch, biliopancreatic limb, roux limb, and two anastomoses, namely, the GJ and JJ anastomoses [6]. This allows caloric restriction and malabsorption as nutrients bypass two-thirds (⅔) of the intestinal system, thus promoting weight loss [6]. RYGB is associated with complications due to manipulation of the anatomy and physiology of the gastrointestinal (GI) tract. The most common presenting feature is abdominal pain, which is a nonspecific symptom [7]. Below are the underlying etiologies of abdominal pain experienced by our patients after the RYGB procedure.

Marginal ulcer

This condition is characterized by the appearance of one or more peptic ulcers on the jejunal mucosa at the gastrojejunal anastomosis [7]. Some notable risk factors for marginal ulcer development include type 2 diabetes, tobacco use, preoperative colonization with H. pylori, and long gastric pouches [8]. This increases acid exposure and leads to mucosal ischemia of the unprotected jejunum [5]. Patients commonly present within three months complaining of epigastric pain, nausea, vomiting, and, in some cases, darkening of the stools due to upper GI bleeding [7]. Endoscopy with visualization of the esophagus, gastric pouch, and Roux limb is the imaging modality of choice [7]. Management with PPIs and repeat endoscopy is utilized to ensure healing, and in 9% of cases, surgical revision may be required [8]. Marginal ulcers may lead to stricture formation and, in some cases, perforation, which is a rare but fatal complication that requires emergent surgery [7]. In the case of complicated marginal ulcers, then anastomosis resection and recreation may be required [8].

Small bowel obstruction

Small bowel obstructions (SBO) are mainly caused by internal hernias, postoperative adhesions, or strictures [7]. Placement of the jejunum in a retrocolic fashion during RYGB creates a mesenteric defect known as Petersen’s Space, which increases the rate of internal hernia occurrence [9]. Petersen’s space is bordered by the roux limb mesentery, transverse mesocolon, and retroperitoneum [9]. This defect allows the small intestine to slide through and become herniated, leading to SBO. Loss of mesenteric fat due to rapid weight loss widens the intermesenteric spaces and can also lead to internal herniation [10]. Antecolic placement of the anastomosis is associated with a decreased risk of internal hernia-induced SBO; therefore, adhesions and strictures are more commonly reported [9]. The most common presenting feature of this condition is crampy abdominal pain with nausea and vomiting [7]. SBO is a very fatal complication that requires prompt diagnosis and treatment to prevent bowel ischemia and necrosis [5]. CT scans and abdominal X-rays are suitable imaging modalities to diagnose SBOs in their early or late stages [11]. Imaging usually shows a dilated proximal bowel loop, a collapsed distal loop, and a transition point where there is delayed passage of intestinal material [11]. Treatment is with either laparoscopic or open diagnostic exploration, with the release of the small bowel and addressing the underlying cause via adhesiolysis, stricture dilation, or closure of the mesenteric defect [6].

Intussusception

Intussusception most commonly occurs at the JJ anastomosis, where the distal common limb telescopes into the proximal jejunal limb [12]. Some studies report that the risk of intussusception is higher in women who have significant weight loss; this infers that presentation may occur several months to years after initial bariatric surgery [5]. The presence of a lead point such as suture lines or adhesions is the main cause; however, in some cases, a non-lead point intussusception may occur [12,13]. Rapid weight loss can also lead to mesenteric thinning, which increases bowel motility and precipitates intussusception [6]. The presentation of this condition can range from asymptomatic to severe, with symptoms of abdominal pain, nausea, and vomiting. Additionally, the characteristic feature of bloody stools is not always seen in post-bariatric patients [12]. Imaging with a CT scan is the diagnostic modality of choice, which shows the classic "target sign," characterized by alternating areas of echogenicity and hypoechogenicity [6]. The gold standard surgical mode of management is laparoscopic reduction; some alternatives include resection with revision of the anastomosis or reduction and enteropexy [12].

In our case, both patients who exhibited immense weight loss presented with SBO, intussusception, and marginal ulcers. Diagnostic testing with CT, abdominal X-ray, and EGD was utilized to come to a timely diagnosis in both patients and allow early intervention. A close follow-up with these patients is necessary to prevent recurrence and the development of complicated cases.

## Conclusions

RYGB can present with several complications, and timely management is important in the treatment of patients. In the cases presented, both patients had similar presentations with the common complaint of abdominal pain, but diagnostic imaging revealed different etiologies. One patient had SBO, the other had intussusception, and both were found to have marginal ulcers. Each of these complications has different risk factors, clinical durations, and management strategies, but they all share one common chief complaint: abdominal pain. Therefore, it is imperative that physicians have an understanding of the etiologies and next best steps required when presented with a bariatric patient complaining of abdominal pain post-RYGB.
